# Preemptive use of etodolac on tooth sensitivity after in-office bleaching: a randomized clinical trial

**DOI:** 10.1590/1678-7757-2016-0473

**Published:** 2018-01-16

**Authors:** Savil Costa Vaez, André Luís Faria-e-Silva, Alessandro Dourado Loguércio, Micaelle Tenório Guedes Fernandes, Flávia Pardo Salata Nahsan

**Affiliations:** 1 Universidade Federal de Sergipe, Aracaju, SE, Brasil; 2 Universidade Estadual de Ponta Grossa, Faculdade de Odontologia, Departamento de Odontologia Restauradora, Ponta Grossa, PR, Brasil

**Keywords:** Anti-inflammatory agents, Tooth bleaching, Dentin sensitivity, Drug prescriptions

## Abstract

**Purpose:**

This study determined the effectiveness of the preemptive administration of etodolac on risk and intensity of tooth sensitivity and the bleaching effect caused by in-office bleaching using 35% hydrogen peroxide.

**Material and methods:**

Fifty patients were selected for this tripleblind, randomized, crossover, and placebo-controlled clinical trial. Etodolac (400 mg) or placebo was administrated in a single-dose 1 hour prior to the bleaching procedure. The whitening treatment with 35% hydrogen peroxide was carried out in two sessions with a 7-day interval. Tooth sensitivity was assessed before, during, and 24 hours after the procedure using the analog visual scale and the verbal rating scale. Color alteration was assessed by a bleach guide scale, 7 days after each session. Relative risk of sensitivity was calculated and adjusted by session, while overall risk was compared by the McNemar's test. Data on the sensitivity level of both scales and color shade were subjected to Friedman, Wilcoxon, and Mann-Whitney tests, respectively (α=0.05).

**Results:**

The preemptive administration of etodolac did not affect the risk of tooth sensitivity and the level of sensitivity reported, regardless of the time of evaluation and scale used. The sequence of treatment allocation did not affect bleaching effectiveness, while the second session resulted in additional color modification. The preemptive administration of etodolac in a single dose 1 hour prior to in-office tooth bleaching did not alter tooth color, and the risk and intensity of tooth sensitivity reported by patients.

**Conclusion:**

A single-dose preemptive administration of 400 mg of etodolac did not affect either risk of tooth sensitivity or level of sensitivity reported by patients, during or after the in-office tooth bleaching procedure.

## Introduction

Tooth whitening is a simple and non-invasive treatment commonly carried out to reestablish smile aesthetics. High success rates have been demonstrated for bleaching techniques applying 35% hydrogen peroxide (HP35%)^2^. Hydrogen peroxide (H_2_O_2_)-based bleaching agents at high concentrations (typically 15-38%) are currently used for in-office techniques due to their high oxidizing ability^12^ ^,^ ^25^. However, the low molecular weight of H_2_O_2_ allows its penetration across the entire dentin tissue, reaching the pulp chamber and promoting damage of pulp stem cells, which is reported by patients as tooth sensitivity^15^ ^,^ ^29^.

Prior clinical trials have reported absolute risk of tooth sensitivity as high as 95% when highly concentrated H_2_O_2_ is used for in-office tooth bleaching^9^ ^,^ ^20^ ^,^ ^23^ ^,^ ^25^. Thus, the preemptive use of desensitizer agents^3^ ^,^ ^30^ or anti- inflammatories^7^ ^,^ ^22^ ^,^ ^23^ ^,^ ^27^ has been proposed to reduce the risk of post-bleaching tooth sensitivity. Only the former significantly decreased tooth sensitivity; however, the application of desensitizers, when not incorporated into the bleaching gel, adds an extra step to the bleaching protocol, which is contrary to a clinician's need for simplification. On the other hand, the preemptive use of anti-inflammatories does not increase the number of steps in the bleaching protocol. Unfortunately, prior studies evaluating etoricoxib, ibuprofen, or dexamethasone were unable to demonstrate any beneficial effect on tooth sensitivity caused by tooth bleaching^7^ ^,^ ^13^ ^,^ ^22^ ^,^ ^23^ ^,^ ^28^.

Etoricoxib and ibuprofen are grouped as class II drugs by the Biopharmaceutical Classification System (BCS), presenting low solubility and high permeability, which can hinder their absorption and create bioavailability mismatch during the bleaching procedure^28^. Moreover, anti-inflammatory drugs may have a specific action over inflammatory mediators (bradykinin) and the neurotransmitter (substance P) of tooth pain caused by dental bleaching^6^. Among non-steroid anti-inflammatory drugs (NSAIDs), etodolac demonstrated efficacy on the control of prostaglandins and bradykinins^18^. Nevertheless, there is no clinical evidence regarding the use of etodolac on the reduction of tooth sensitivity caused by in-office bleaching.

Thus, this study aimed to evaluate the effectiveness of the preemptive prescription of etodolac on risk of tooth sensitivity during and after in-office bleaching treatment. The first hypothesis evaluated was that etodolac would reduce both level and risk of tooth sensitivity when administrated in a single dose prior to in-office bleaching. The second hypothesis tested was whether the use of etodolac would reduce tooth sensitivity with no effect on tooth bleaching.

## Material and methods

This clinical trial followed the CONSORT statements and was approved by the Scientific Review Committee and by the Committee for the Protection of Human Subjects of the local university (CAAE 37578714.4.0000.5546), and registered at clinicaltrials.gov under the number NCT02881619.

### Trial design

This study was a randomized, triple-blind, placebo- controlled clinical trial with a crossover design. Patients included signed an informed consent form and were submitted to two in-office bleaching sessions receiving placebo (control) or etodolac prior to the bleaching procedure, while different treatments were allocated for each session.

A one-week interval ("washout") in-between sessions was established. The study was conducted at the clinic of the School of Dentistry of the local university from November 2014 to July 2015.

### Participants

Patients from 18 to 35 years old with good oral health were included in this clinical trial. From the patients who received placebo/etodolac, 6 were men and 19 were women, and for etodolac/placebo, 12 were men and 13 were women; the average age was 23 years; 64% were women.

Patients with any of the six upper anterior teeth with caries, restoration, severe discoloration (e.g., stains caused by tetracycline), enamel hypoplasia, gingival recession, dentin exposure, pulpitis, or endodontics were excluded, as well as smokers. Participants submitted to previous bleaching procedures, with prior tooth sensitivity, known allergy to any component of the medication used in the study, and pregnant or breastfeeding women were also excluded. An air stream was applied to teeth to verify the presence or absence of sensitivity (none or zero). Only patients presenting all six upper anterior teeth with shade mismatch of 2.5 M2 (Vita Bleach guide 3D-Master scale, Vita-Zahnfabrik, Bad Sackingen, Germany) - A3.5 Vita Classical equivalence - or darker were included.

### Sample size calculation

The sample calculation was based on the primary binary outcome (sensitivity risk 24 hours after the procedure) for superiority trial. The power of the test was set at 80%, considering a type I error of 0.05 and risk of tooth sensitivity of 90%, based on a prior study using a similar bleaching agent^23^; moreover, a reduction around 30% was expected after treatment. The calculation resulted in fifty patients.

### Randomization

A randomized list was computer-generated by a person not involved in intervention or evaluation. Participants were defined as blocks in the randomization process, in which the sequence of treatment (placebo or etodolac) was randomly set for each block by computer-generated tables (www.sealedenvelope.com). The sequence was inserted into sealed envelopes numbered from 1 to 50 that were opened by the operator only at the moment of intervention. Patients were numbered according to the sequence of enrollment. Neither the participant nor the operator knew the group allocation, determining a blinded protocol.

### Baseline evaluation

Prior to the bleaching procedure, teeth were cleaned using rubber cups associated with pumice and water. The shades of upper incisors and canine teeth (13, 12, 11, 21, 22, and 23) were assessed on a baseline using the bleach guide scale. Color was analyzed by two previously calibrated evaluators. Both evaluators presented superior color matching competency according to the ISO (International Organization for Standardization)/TR 28642:2011. Shade tabs selected were converted to scores ranging from 1 (whiter shade – 0M1) to 15 (darker shade – 5M3).

Considering a possible effect of dental anxiety on the sensitivity reported by patients, the Corah's Dental Anxiety Scale was used to determine the level of anxiety of each patient related to the procedure^17^. Each answer to the survey instrument was scored on a scale from 1 to 5 (four questions) and the sum of scores was used to determine the level of anxiety: low was under 12, moderate was between 12 and 14, and high was over 14.

### Intervention

One hour before each bleaching session and right after the prophylaxis, patients received a capsule containing 400 mg of NAISE etodolac (Flancox™, Apsen Farmaceutica S/A) or 400 mg of placebo (inert content) according to randomization. Capsules had the same appearance and were manufactured by a person not involved in intervention or evaluation. They were placed into two bottles identified by letters according to the treatment. Neither the operators responsible for intervention and evaluation nor the patients knew the content of each capsule.

The color evaluation was verified, and the light- cured resin dam was applied (Top Dam, FGM, Joinville, SC, Brazil) and polymerized (Radii-cal, SDI, Bayswater, Australia) on the gingival tissue corresponding to the teeth to be bleached. A 35% hydrogen peroxide-based bleaching agent (Whiteness HP Blue, FGM, Joinville, SC, Brazil) was mixed and applied to the buccal surface of teeth for 40 minutes. After that, the bleaching agent was removed. A second session was carried out after a week following the same procedures. At this time, the patient received a single-dose capsule containing etodolac or placebo (different from the ones received at the first session), one hour before the procedure. During the bleaching treatment, patients were advised not to ingest colored food and beverages.

### Evaluations

Tooth sensitivity reported by patients was assessed using a visual analog scale (VAS) and a verbal rating scale (VRS). The VAS consisted of a 10-cm long scale ranging from green (absence of pain) to red (unbearable pain). Patients set their level of sensitivity by pointing to the color corresponding to the pain level, while the distance from this point to the green border was recorded. For the VRS, patients reported their level of sensitivity based on scores: 0= none; 1 = mild; 2= moderate; 3= considerable; and 4= severe. Tooth sensitivity was assessed during bleaching, immediately after removing the bleaching agent, and after 24 hours. For this last assessment, only the VRS was used due to the difficulty of patients to fill the VAS at home. One week after each session, tooth color was evaluated again using the same procedure described previously.

### Statistical analysis

Demographic data from patients were analyzed to determine age, gender, and anxiety level for each allocation sequence. Comparisons among allocation sequences were performed by Mann-Whitney (age), Fisher's exact (gender), and chi-square (anxiety level) tests.

Based on the presence of any tooth sensitivity (VRS scores different from 0), the absolute risk, odds ratio, and relative risk were calculated regarding the treatments for each moment of bleaching evaluation/ session, as well as the confidence intervals (95%). For each moment, differences on presence/absence ratios were analyzed by Fisher's exact test. For the overall risk related to each treatment, odds ratio was adjusted to the independent variable of "session of bleaching" using Mantel-Haenszel statistics. The homogeneity of odds ratios was analyzed by Breslow-Day and Tarone's tests. Next, the estimated odds ratio was converted to relative risk and the overall presence/absence ratios were analyzed by the McNemar's test, considering the study design (crossover).

For the VRS, data from scores observed at each moment of bleaching evaluation/session were submitted to the Mann-Whitney rank sum test. Despite the measurement of tooth sensitivity using the VAS, which provided a continuous outcome, data assessed with this scale did not show normal distribution (Shapiro-Wilk's test). Thus, data from the VAS were also analyzed by the Mann-Whitney rank sum test, performing one test per time of evaluation.

For color evaluation, comparisons among sequences of treatment were performed using the Mann-Whitney rank sum test. Friedman test followed by Dunn's *post hoc* test were used to analyze the difference between the moments of evaluation for each sequence of treatment. All statistical analyses were performed adjusting the initial significance level (α=0.05) by the Bonferroni correction.

## Results

**Figure 1:**
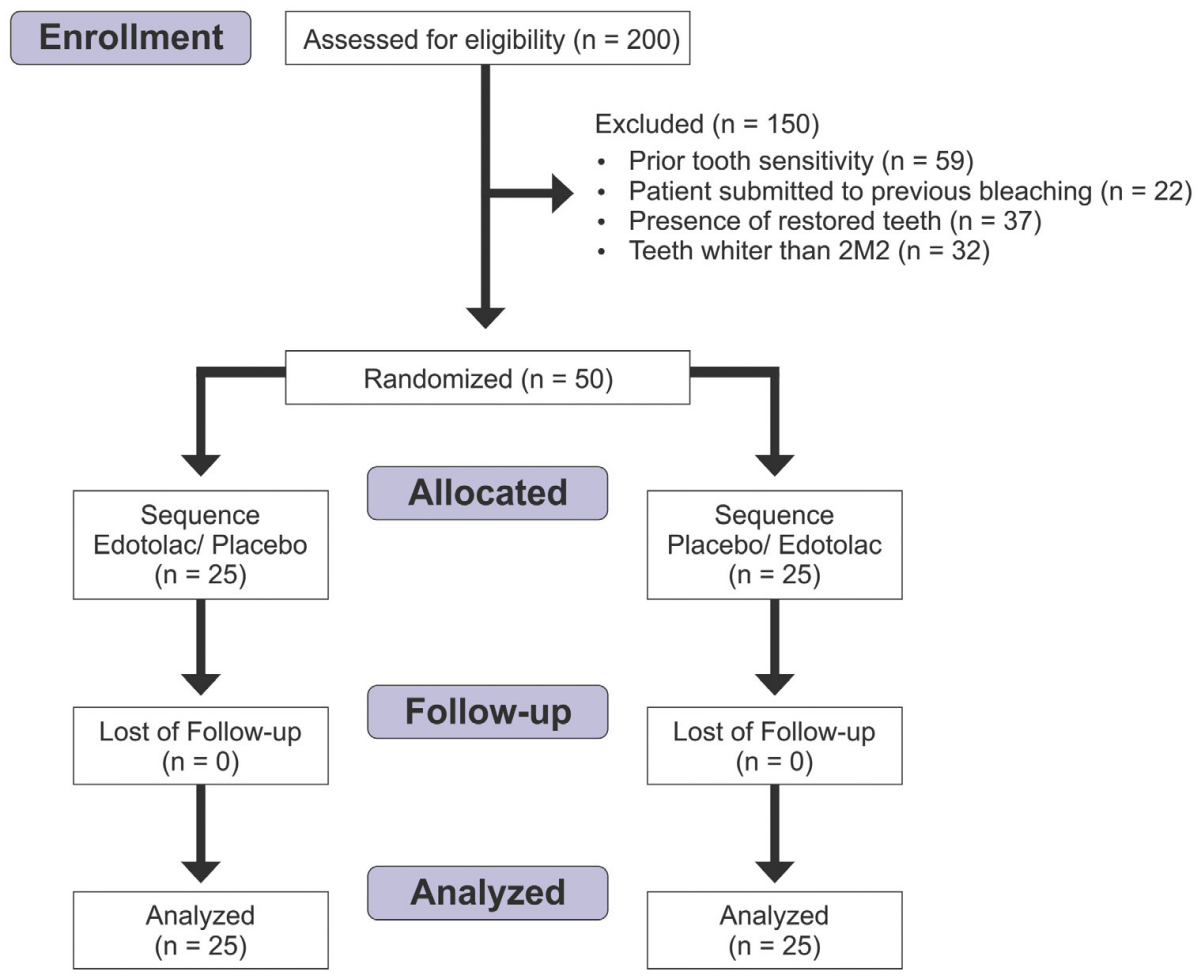
Flow chart of patients

Figure 1 shows the flow chart of patients assessed for eligibility, who were included in the study and analyzed. Table 1 shows the demographic characteristics of patients allocated for each sequence of treatment. Regarding anxiety, 88% of patients had a low level and only 2% had a high level (p=0.236). There was no difference among the sequences of treatment for any demographic characteristic analyzed (age: p=0.089 and gender: p=0.140).

**Table 1:** Profile of patients included in the study allocated to each sequence of treatment

Age (years)		Median (1^st^ / 3^rd^ quartiles)	p-value
**Total**		**23.0 (21.0/26.0)**	
Placebo/Edotolac		23.8 (21.0/25.5)	**p** * **= 0.089**
Edotolac/Placebo		25.0 (22.0/28.0)	
**Gender Total**		**n (%)**	

	Male	18 (36.0%)	
	Female	32 (64.0%)	
Placebo/Edotolac			**p** ** **= 0.140**
	Male	6 (24.0%)	
	Female	19 (76.0%)	
Edotolac/Placebo			
	Male	12 (52.0%)	
	Female	13 (48.0 %)	
**Level of anxiety Total**		**n (%)**	

	Low anxiety	44 (88.0%)	
	Moderate anxiety	5 (10.0%)	
	High anxiety	1 (2.0%)	
Placebo/Edotolac			**p** *** **= 0.236**
	Low anxiety	21 (84.0%)	
	Moderate anxiety	4 (16.0%)	
	High anxiety	0 (0.0%)	
Edotolac/Placebo			
	Low anxiety	23 (92.0%)	
	Moderate anxiety	1 (4.0%)	
	High anxiety	1 (4.0%)	

* Mann-Whitney rank sum test;

** Fisher Exact test;

*** Chi-square test.

**Figure 2:**
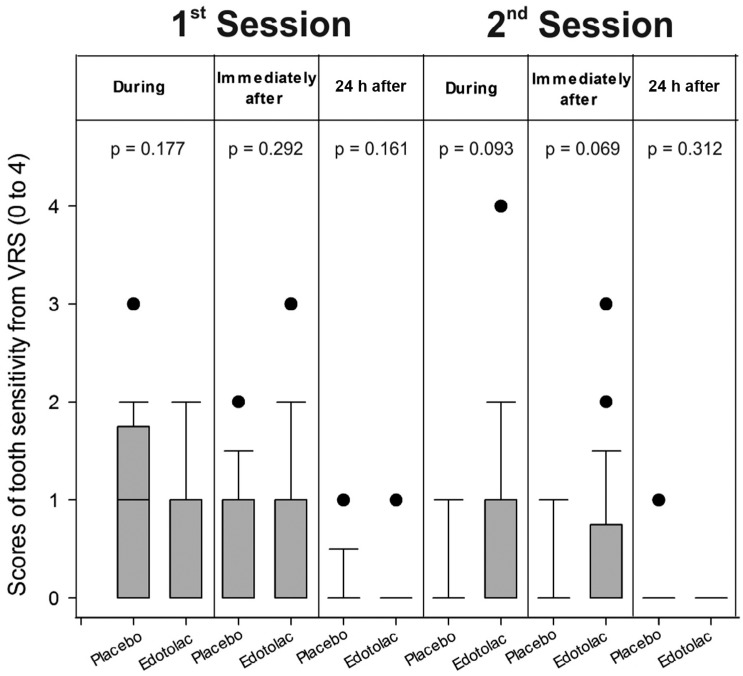
Scores of tooth sensitivity from the VRS (0 to 4)

**Figure 3:**
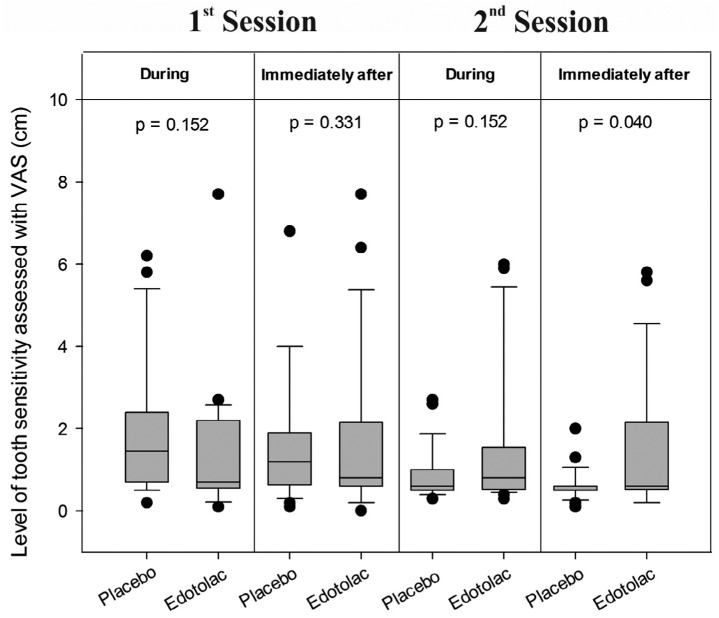
Level of tooth sensitivity assessed with VAS (cm)

Table 2 shows the results of tooth sensitivity risk. The treatment that patients received prior to the bleaching procedure did not affect the risk to sensitivity at any of the moments of evaluation (during and immediately after: p=1.0; after 24 hours: p=0.683). Figure 2 shows the results for level of sensitivity assessed by the VRS. Treatment did not affect level of sensitivity, regardless of the moment of evaluation. Similar results were observed when the VAS was used (Figure 3).

**Table 2:** Results of risk of tooth sensitivity observed for each treatment

Session	Moment of evaluation	During		Immediately after		24 h after	
	Treatment	Edotolac	Placebo	Edotolac	Placebo	Edotolac	Placebo
1^st^ session	Presence of sensitivity (yes/no)	(10/15)	(11/14)	(9/16)	(10/15)	(3/22)	(2/23)
	Odds ratio (95% CI)	0.52 (0.17 – 1.61)		0.84 (0.27 – 2.65)		1.57 (0.24 – 10.30)	
	Relative risk (95% CI)	0.71 (0.40 – 1.29)		0.90 (0.44 – 1.83)		1.50 (0.27 – 8.22)	
	p-value*	0.396		1.000		1.000	
2^nd^ session	Presence of sensitivity (yes/no)	(8/17)	(5/20)	(6/19)	(4/21)	(1/24)	(1/24)
	Odds ratio (95% CI)	1.88 (0.52 – 6.85)		1.66 (0.41 – 6.79)		1.00 (0.59 – 16.93)	
	Relative risk (95% CI)	1.60 (0.61 – 4.22)		1.50 (0.48 – 4.68)		1.00 (0.06 – 15.12)	
	p-value*	0.520		0.725		1.000	
Average	Odds ratio (95% CI)**	0.92 (0.40 – 2.09)		1.11 (0.46 – 2.67)		1.37 (0.29 – 6.51)	
	Relative risk***	0.95		1.08		1.34	
	p-value****	1.000		1.000		0.683	

* Fisher exact test;

** Mantel-Haenszel common odds ratio estimate;

*** Based on odds ratio estimated;

**** McNemar's test. The cut-off value of type I error (α=0.0056) was adjusted by Bonferroni correction.

**Figure 4:**
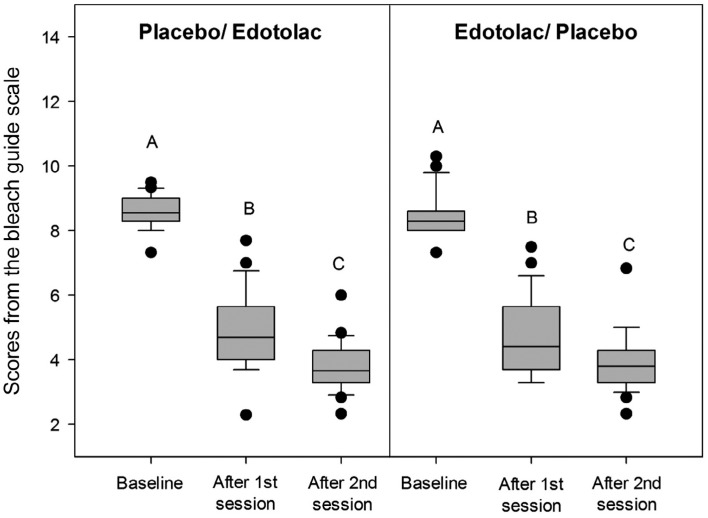
Scores from the bleach guide scale

In regards to bleaching effectiveness, the bleaching procedure carried out in this study was able to significantly reduce the shade scores from the bleach guide, while the second session resulted in additional bleaching effect (Figure 4). The sequence of treatment did not affect bleaching effectiveness.

## Discussion

Tooth bleaching performed by patients at home using low-concentration peroxides has been reported as the first-choice technique for vital bleaching, and probably the most widely used one^5^ ^,^ ^11^. However, procedures using high-concentration bleaching agents applied in office by clinicians remain an important protocol used to bleach discolored teeth for specific indications of tooth bleaching. This protocol is separate and intended to aid patients who cannot adapt to the use of home-based bleaching trays and who have contraindications related to the gastric system, because it reduces the risk of gel intake^14^, and to control the risk factor for developing gingival irritation^10^. In-office bleaching is also indicated to patients requiring faster results, while this technique can be combined with at-home bleaching^26^.

In-office techniques have demonstrated high bleaching effectiveness using high-concentration hydrogen peroxides^26^. However, high-concentrated bleaching agents also result in increased tooth sensitivity reported by patients during and up to 24 hours after the bleaching procedure, which is the main adverse effect related to in-office tooth bleaching^9^ ^,^ ^20^ ^,^ ^21^. Even though tooth sensitivity is related to the inflammatory process of pulp tissue^20^, the findings of this study showed that the preemptive use of etodolac in a single dose did not affect the risk and level of tooth sensitivity caused by in-office bleaching. As expected, preemptive administration of etodolac also did not affect the bleaching results. Thus, the first hypothesis of the study was rejected and the second one was accepted.

Unlike the tooth sensitivity typically reported by patients presenting teeth with dentin exposure, which relates mainly to thermal stimuli, bleached teeth can hurt even in the absence of any stimulus, showing that the pain mechanism related to peroxides is different from other types of tooth pain^20^. Moreover, the sensitivity caused by tooth bleaching tends to increase within a few hours following the bleaching procedure, when most patients described the pain as a "twinge" or "shock-like." It has been demonstrated that the oxidizing agents used during the bleaching procedure cause a reduction on metabolism, viability, and cell proliferation^20^, allowing to increase the expression of inflammatory mediators, such as substance P and bradykinin, which is a vasoactive peptide released by nerves resulting in a neurogenic inflammation^6^. Thus, a preemptive administration of anti-inflammatories could be a reasonable approach to reduce tooth sensitivity associated with bleaching procedures. However, in this study, sensitivity was not lower during and 24 hours after the bleaching procedure. Unfortunately, the peak of tooth sensitivity was not measured in this study, even though this outcome could allow to assess a possible effect of etodolac on sensitivity following the end of the bleaching procedure.

A prior study demonstrated that etodolac presents higher effectiveness on bradykinin inhibition than other drugs commonly used to control the inflammatory process, motivating its use in this study^18^. Regarding pharmacokinetics, etodolac reaches its maximal plasma concentration around 1 to 2 hours after its administration^4^. Thus, it was expected that maximal plasma concentration would be reached during the bleaching procedure. Unfortunately, no effect of the preemptive administration of etodolac on the reported tooth sensitivity was observed at this time of evaluation for both sessions. Despite its demonstrated efficacy on inflammation control, the analgesic effect of etodolac increases when its administration is repeated, whereas a single dose prior to the bleaching procedure seems insufficient to prevent tooth sensitivity^19^.

Another important observation regarding pharmacokinetics is that a crossover design was used in this study with a 1-week interval period. Considering that the half-life of etodolac after oral administration is around 13 hours^16^, it is expected to find no residual effect after 1-week. Moreover, the crossover design avoids bias related to pain thresholds of patients^1^.

Participants included in this study were predominantly young females presenting low level of anxiety. All these demographics characteristics of the studied population might be associated with differences in pain thresholds. Higher tooth sensory threshold has been demonstrated in males due to differences in crown diameters of teeth and underlying mechanisms such as neurological differences or behavior aspects^8^. Regarding the age of participants, a recent review did not find any relation between age and the risk to or level of tooth sensitivity^27^. However, it is important to emphasize that most participants from trials included in that review were under 30 years old^27^. Another important demographic aspect assessed in this study was the participants' level of dental anxiety prior to bleaching procedures. It has been demonstrated that dental anxiety is a strong predictor of pain and that anxious participants are prone to develop painful responses^24^. In this research, almost 90% of participants presented low anxiety prior to bleaching procedures, which can be justified by the low invasive aspect of intervention, despite the patients' concern about tooth sensitivity. In fact, despite 66% of participants reporting various level of tooth sensitivity (high risk), the actual level of sensitivity reported was low (medians below moderate at VRS, and means lower than 2 at VAS).

In addition to the evaluation of tooth sensitivity, we also assessed color alteration promoted by bleaching procedures. The data analysis of color evaluation used the sequence of allocation instead of the treatment (placebo or etodolac). If we had used treatment, different participants between the first and second sessions would be compared for the same treatment, which would have impaired correct color changes assessment. Moreover, the main aim of color evaluation was to show the effectiveness of the bleaching technique used.

In our study, the protocol that was carried out resulted in significant bleaching effect, while an additional color alteration was achieved at the second session. In addition, the last color evaluation was performed a week after the last bleaching session of this study, while longer times may be required for color stabilization^21^. However, a shorter time was used because the tooth sensitivity reported by patients was the main outcome of this trial. It has been demonstrated that two sessions of in-office tooth bleaching results in a mean change of 5.3 (± 2.8) units on shade guides^26^, which is similar to the average color change achieved in this research. Factors such as patient's age and color at baseline have been strongly associated with bleaching effectiveness, while young patients and darker teeth show more pronounced color changes^27^. We found no difference in baseline regarding these parameters between the sequence of allocation, while the inclusion of young participants presenting all teeth darker than 2.5 M2 (score 7) favored obtaining significant color bleaching.

In conclusion, the preemptive administration of etodolac in a single dose 1 hour prior to the bleaching procedure was unable to reduce both risk and level of sensitivity caused by in-office bleaching. A limitation of this study was that the preemptive treatment was administrated only for a young population (average age of 23 years), with prevalence of female patients, while different results can be observed for other demographic profiles^27^.

## Conclusions

The preemptive administration of a single dose of etodolac previously to the two bleaching sessions with 35% hydrogen peroxide did not affect tooth color change, risk of sensitivity and level of pain reported by the patients (during the sessions, immediately after, and 24 h after sessions).
